# *Foxr1* deletion causes microcephaly and leads to cortical and hippocampal hypoplasia

**DOI:** 10.3389/fnins.2025.1589043

**Published:** 2025-05-27

**Authors:** Hannah Waxman, Marcus Kankkunen, Arya Gupta, Margo Dowgiewicz, Uwe Beffert, Angela Ho

**Affiliations:** Laboratory of Dr. Angela Ho, Department of Biology, Boston University, Boston, MA, United States

**Keywords:** Foxr1, transcription factors, microcephaly, cortex, mouse

## Abstract

Foxr1 is a member of the evolutionarily conserved forkhead box (Fox) family of transcription factors, characterized by a winged-helix DNA-binding domain. We previously demonstrated that *Foxr1* deletion in mice results in severe perinatal lethality, cortical thinning, and ventricular enlargement, indicating its essential role in survival and brain development. Here, we extend these findings by showing that *Foxr1* knockout mice develop microcephaly accompanied by cortical and hippocampal hypoplasia at postnatal day 0 (P0). Cortical thinning is primarily driven by a significant reduction in layer 2/3 neurons, linked to impaired generation of later-born neurons. This reduction correlates with decreased proliferation of progenitors (Ki67- and Tbr2-positive cells) at embryonic day 16.5 (E16.5), a critical period for upper-layer neurogenesis. In the hippocampus, *Foxr1* knockouts exhibit reduced area, and cell counts at P0, accompanied by increased proliferation (Ki67-positive cells), and elevated apoptosis (CC3-positive) at E16.5, suggesting broader disruptions in progenitor maintenance. Together, these findings suggest Foxr1 is an important regulator of progenitor dynamics and neuron production in cortical and hippocampal development.

## 1 Introduction

The forkhead box (FOX) family of transcription factors (TFs) were first identified in *Drosophila*, named after the fork-like ectopic head structure observed in forkhead mutants (Weigel et al., 1989). In mammals, FOX proteins have been classified into 19 subfamilies, designated FOXA to FOXS, based on sequence similarity (Larroux et al., 2008). While all FOX proteins share an evolutionarily conserved DNA-binding forkhead domain, sequence variation in other regions confers distinct regulatory properties across subfamilies, enabling FOX factors to govern diverse biological processes including embryonic development, organogenesis, cell cycle control, stem cell maintenance, and signal transduction (Lee et al., 2005; Halasi and Gartel, 2013; Li et al., 2015).

Human FOXR1 encodes a 292 amino acid protein, sharing 77.4% sequence identity with rat (Katoh and Katoh, 2004b). Phylogenetic analysis indicates that Foxr1 is conserved across vertebrates. In zebrafish, *Foxr1* acts as maternal-effect gene necessary for early embryonic viability (Cheung et al., 2018), while in mice, *Foxr1* mRNA is detected in embryonic germ cells and fertilized eggs (Katoh and Katoh, 2004a). Human tissue expression data from GTEx shows *FOXR1* expression in the brain, testis, and ovary, with ubiquitous expression across all brain regions throughout postnatal development according to the Human Brain Transcriptome. Supporting the potential significance of FOXR1 in the brain, the NIH Undiagnosed Disease Network identified an individual with a heterozygous FOXR1 variant presenting severe neurological symptoms including postnatal microcephaly, progressive brain atrophy, and global developmental delay (Mota et al., 2021). To explore FOXR1 function, we previously overexpressed human FOXR1 in HEK293T cells and performed RNA-sequencing analysis, revealing both up- and down-regulated genes compared to control. Notably, the top upregulated genes were enriched for protein-folding chaperones including heat shock response genes and pathways involved in suppressing inclusion body formation, suggesting a potential role for FOXR1 in cellular stress regulation (Mota et al., 2021). Using CRISPR/Cas9 gene editing, we found that deletion of mouse *Foxr1* leads to severe survival deficits (Mota et al., 2021). Newborn *Foxr1* knockout brains display a decrease in cortical thickness and enlarged ventricles, suggesting that loss of *Foxr1* leads to atypical brain development. However, these findings only hint at the critical, yet poorly understood function of Foxr1 in early brain development.

Here, we demonstrate *Foxr1* deletion results in microcephaly, cortical thinning, and hippocampal hypoplasia. The cortical thinning in layer 2/3 is driven, in part, by reduction in intermediate progenitor proliferation of later-born neurons at E16.5. In the hippocampus, *Foxr1* knockout mice display reduced size with increased progenitor proliferation and elevated apoptosis at E16.5. These findings suggest Foxr1 is an important regulator of progenitor cell dynamics and neuron production during cortical and hippocampal development.

## 2 Materials and methods

### 2.1 Animals

The *Foxr1* (C57BL/6N-Foxr1<em1(IMPC)Tcp>) mouse line was generated using CRISPR/Cas9 gene editing at The Centre for Phenogenomics, Canada, and obtained from the Canadian Mouse Mutant Repository. Genotyping and knockout generation methods were previously described (Mota et al., 2021). All animal procedures were approved by the Boston University Institutional Animal Care and Use Committee, and methods were conducted in accordance with institutional and national guidelines and regulations.

### 2.2 Tissue processing and histological analysis

Newborn and embryonic mouse brains were fixed in 4% paraformaldehyde (PFA) in phosphate-buffered saline (PBS) and cyroprotected in 5% sucrose in PBS (overnight for newborn, and 6–8 h for embryos). Brains were then sequentially dehydrated in 10, 20 and 30% sucrose in PBS at 4°C for 12 h each. Next, brains were incubated in graded mixtures of 30% sucrose:OCT embedding compound (Fisher NC9636948) (2:1, 1:1, and 2:1) for 25 min each at room temperature (RT), followed by 100% OCT for 20 min. Brains were frozen in 2-methylbutane on dry ice, cryosectioned (20 μm), mounted on SuperFrost microscope slides (Fisher 12-550-12) and stored at −80°C. For Nissl staining, sections were dried for ~6 h at RT, stained with 1% cresyl violet solution for 2.5 min and dehydrated through a graded ethanol series: ddH_2_O, ddH_2_O, 50, 70, 95, and 100% EtOH, 5 s each, followed by clearing in xylenes. Slides were mounted with Permount media (Fisher SP15-100) and dried overnight. For immunofluorescences, sections were thawed for 25 min, rehydrated in PBS for 20 min, and processed using one of three antigen retrieval protocols (Sodium Citrate, HistoVT or HistoVT+). Sodium citrate: sections were treated with 0.3% methanol peroxidase for 10 min, rinsed with PBS and microwaved in 10 mM sodium citrate buffer pH 6.0 at 900 W for 90 s then 100 W for 10 min followed by PBS washes. HistoVT: sections were incubated in pre-warmed HistoVT One (Nacalai USA NC0499519) at 70°C for 30 min, then cooled. After antigen retrieval, sections were washed and blocked (5% goat serum, 0.3% Triton X-100 in PBS) for 1 h at RT followed by primary antibody in 5% goat serum in PBS at 4°C overnight. Sections were then washed and incubated with secondary antibody in 5% goat serum in PBS for 1 h at RT, washed and mounted with Prolong Gold (cat# P36931 Fisher). For HistoVT+, both primary and secondary antibodies were incubated in blocking solution with primary antibody incubated overnight at RT. Antibodies and dilutions are provided in Supplementary Table 1. Images were captured using a Zeiss LSM 700 confocal microscope with a 25× objective via tile scan in a single focal plane with post-acquisition stitching.

### 2.3 Quantification and statistical analysis

Experimenters were blinded to conditions during data analysis. At P0, whole brain length (cortex to cerebellum, excluding olfactory bulbs and brainstem) and cortical width (widest point) were measured using the line tool in FIJI. Total brain area, cortex, and tectum/cerebellum were traced with the freehand selection tool. Cortical layer thickness was measured using DAPI staining (layers 1 and 2/3), Tbr1 immunostaining (layers 5/6) and subtraction to estimate layer 4 (measured with the FIJI line tool). Cell counts (DAPI, Satb2, or Tbr1 positive cells) were performed manually using the FIJI/ImageJ Cell Counter plugin. Satb2-positive cells were quantified within layer 2/3 and Tbr1-positive cells in layer 5/6. CC3-positive apoptotic cells, Iba1-positive microglia, and Ki67-positive proliferating cells were quantified in the ventricular zone (VZ) using 100 μm × 200 μm bins, with whole-slice microglia counts automated via the Automorfi plugin (Bouadi et al., 2024). Hippocampal cell counts were performed with CellProfiler (Bouadi and Tay, 2024). Lateral corpus callosum (CC) thickness was measured from ventricle top to cortical plate and medial CC at the midline using the FIJI line tool. The hippocampus, striatum, and cerebellum were outlined with the freehand tool to calculate cross-sectional areas. All statistical parameters are expressed as means ± SEM. Unpaired Student’s *t*-tests were used for pairwise comparisons, and one-way analysis of variance (ANOVA) with Tukey’s *post-hoc* test for multiple comparisons, and two-way ANOVA with Sidak’s *post-hoc* test for multiple comparisons were performed using Prism 9 software (GraphPad). Number of animals and statistical information are reported on the corresponding figure legends.

## 3 Results

### 3.1 Loss of *Foxr1* causes microcephaly in surviving neonate pups

**Figure 1:**
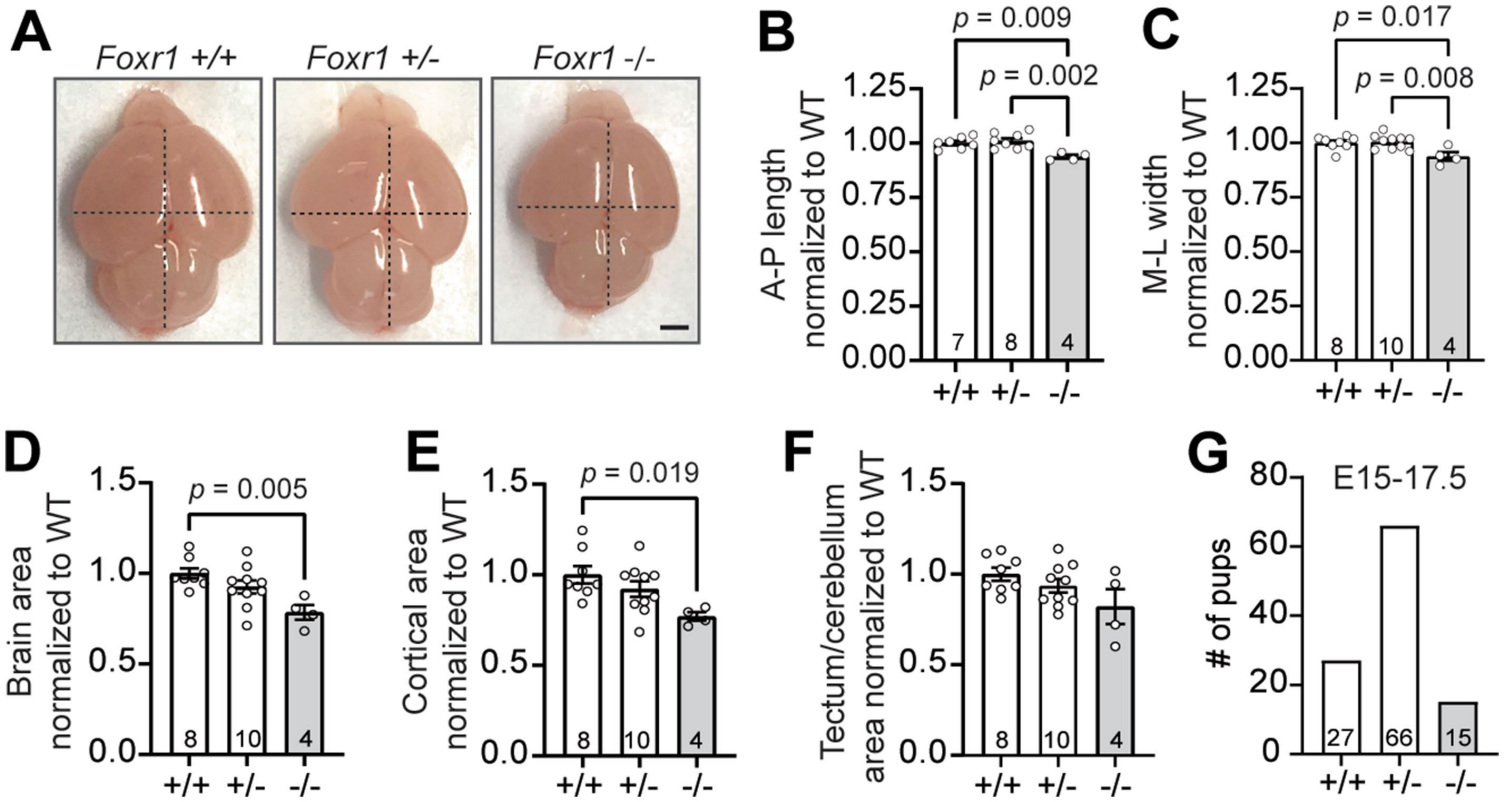
*Foxr1* knockout mice exhibit microcephaly primarily affecting the cortex. **(A)** Representative dorsal images of unfixed P0 brains from *Foxr1* wildtype (*+*/*+*), heterozygous (+/−) and knockout (*−*/*−*) mice. Dashed black lines indicate anterior-posterior (A-P) length and medio-lateral (M-L) width measurements. Scale bar = 1 mm. **(B–F)** Quantification of brain size metrics, normalized to *Foxr1* wildtype (WT) values. **(B)** Anterior-posterior (A-P) brain length: *p* = 0.009 (+/+ vs. −/−); *p* = 0.002 (+/− vs. −/−). **(C)** Medio-lateral (M-L) brain width: *p* = 0.017 (+/+ vs. −/−); *p* = 0.008 (+/− vs. −/−). **(D)** Total brain area; *p* = 0.005 (+/+ vs. −/−). **(E)** Cortical brain area: *p* = 0.019 (+/+ vs. −/−). **(F)** Tectum/cerebellar brain area: not significant. **(G)** Genotype distribution of embryos collected from *Foxr1* heterozygous crosses between E15–17.5. Sample sizes (indicated in bars) represent individual animals. Statistical significance was determined using one-way ANOVA with Tukey’s multiple comparisons test. Error bars indicate SEM.

We previously reported that the majority of *Foxr1* knockout mice do not survive postnatal development, and those that do are smaller and weigh less compared to their littermates at birth (Mota et al., 2021). One striking phenotypic difference observed in *Foxr1* knockout (−/−) neonates is a significant reduction in brain size at postnatal day 0 (P0) compared to wildtype (+/+) littermates, as shown by dorsal imaging for gross morphological assessment (Figure 1A). Quantitative analysis of cortical dimensions at P0 showed a 6.3% reduction in the anterior-to-posterior (A-P) axis in *Foxr1* knockout mice compared to both wildtype and heterozygous (+/−) littermates (*F*_2,16_ = 8.895, *p* = 0.009 wildtype vs. knockout; *p* = 0.002 heterozygous vs. knockout, Figure 1B). Similarly, the medial-to-lateral (M-L) axis, measured at the widest part of the forebrain, was decreased by 6.2% in *Foxr1* knockout mice (*F*_2,19_ = 6.253, *p* = 0.017 wildtype vs. knockout; *p* = 0.008 heterozygous vs. knockout, Figure 1C). In addition, overall brain and neocortical areas are reduced by 21.5 and 23%, respectively in *Foxr1* knockout mice compared to controls (*F*_2,19_ = 6.46, *p* = 0.005, Figure 1D; *F*_2,19_ = 4.532, *p* = 0.019, Figure 1E, respectively), while tectum and cerebellar area remains unchanged (Figure 1F). We previously reported that only ~34% of *Foxr1* knockout mice survive to P0. Additional genotyping of embryonic litters reveals substantial attrition by mid- to late gestation (E15–E17.5), with only ~50% of the expected *Foxr1* knockout embryos recovered (*χ*^2^ = 8.0, df = 2, *p* = 0.018, Figure 1G). In 5 of the 14 litters analyzed during this window, no knockout embryos were present. These findings underscore a major limitation of investigating Foxr1 function in a full knockout model, given the high rate of embryonic lethality. Despite this limitation, our findings demonstrate that in the subset of *Foxr1* knockouts that survive to birth, loss of *Foxr1* leads to microcephaly, primarily driven by cortical reduction.

### 3.2 *Foxr1* deletion selectively impairs cortical layer 2/3 development

**Figure 2:**
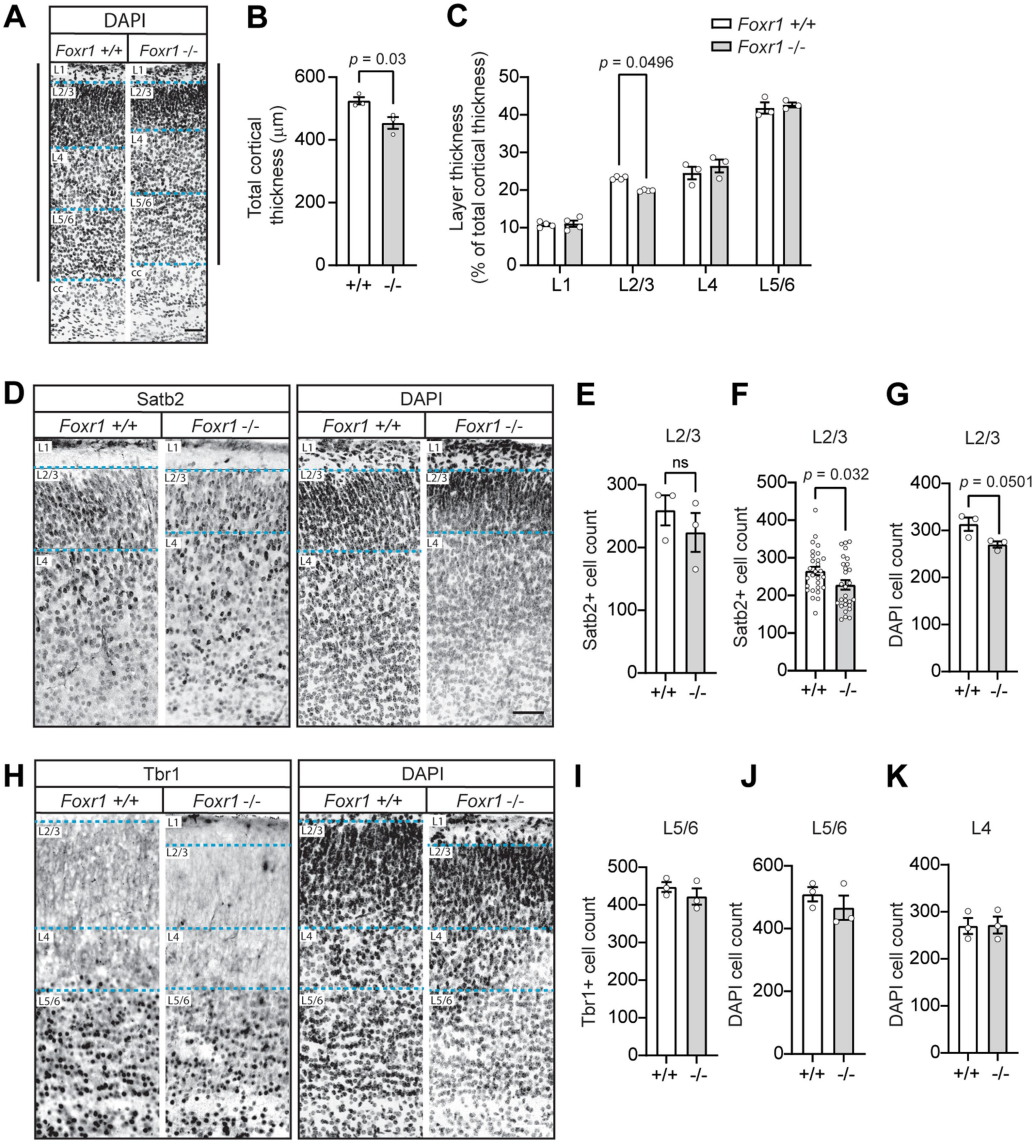
Foxr1 knockout mice exhibit selective thinning and reduced cell density in cortical layer 2/3 at P0. **(A)** Representative DAPI-stained coronal sections from P0 *Foxr1* wildtype (+/+) and knockout (−/−) mice. Solid vertical black lines denote total cortical thickness from the corpus callosum (cc) to the pial surface, and dashed blue lines indicate cortical layer boundaries. **(B)** Quantification of total cortical thickness shows a reduction in *Foxr1* −/− mice (*p* = 0.03). *N* = 3 animals per genotype; ~14 sections per animal. **(C)** Analysis of layer thickness as a percentage of total cortical thickness reveals a selective reduction in layer 2/3 in *Foxr1* −/− mice (*p* = 0.0496). *N* = 3–4 animals per genotype; ~9 sections per animal. **(D)** Representative images of Satb2 immunostaining (left) and corresponding DAPI-stained nuclei (right) in the cortical column. Dashed blue lines indicate cortical layer boundaries. Scale bar = 50 μm. **(E)** Quantification of Satb2-positive cells in layer 2/3, averaged per animal, shows a reduction in *Foxr1* −/− mice that does not reach statistical significance (*p* = 0.421). *N* = 3 animals per genotype; ~8 sections/animal. **(F)** Pooled analysis of Satb2-positive cells in layer 2/3 across all sections (*n* = 28 sections/genotype) shows a significant reduction in *Foxr1* −/− mice (*p* = 0.032) with 3 independent animals per genotype **(G)** Quantification of DAPI-positive nuclei in layer 2/3 shows reduction in *Foxr1* −/− mice (*p* = 0.0501). *N* = 3 animals per genotype; ~8 sections/animal. **(H)** Representative images of Tbr1 immunostaining (left) and DAPI-stained nuclei (right). Dashed blue lines indicate cortical layer boundaries. **(I)** Quantification of Tbr1-positive cells in layer 5/6 shows no difference between genotypes. *N* = 3 animals per genotype; ~10 sections/animal. **(J,K)** Quantification of DAPI-positive nuclei in layer 5/6 **(J)** and layer 4 **(K)** shows no differences. *N* = 3 animals per genotype; ~10 sections/animal. Statistical significance was determined using unpaired Student’s *t*-tests **(B,E–G,I–K)** and two-way ANOVA with Sidak’s multiple comparisons test **(C)**. Error bars represent SEM.

Because the microcephaly in *Foxr1* knockout mice is primarily driven by cortical reduction, we next examined whether this was associated with layer-specific defects in the cortex at P0. To assess this, we examined coronal sections labeled with DAPI to visualize nuclei (Figure 2A). Quantification revealed that cortical radial thickness was reduced by 13.5% in *Foxr1* knockout mice compared to wildtype littermate controls, consistent with our previous findings of cortical thinning (*p* = 0.03, Figure 2B) (Mota et al., 2021). Notably, this thinning was specifically attributed to a 14.5% reduction in layer 2/3 (genotype *F*_1,20_ = 0.021, *p* = 0.886; cortical layer *F*_3,20_ = 376.8, *p* < 0.0001; interaction *F*_3,20_ = 3.145, *p* = 0.0479), whereas deeper cortical layers remained unchanged (*post hoc* pairwise comparison layer 2/3, *p* = 0.0496, Figure 2C). To determine whether the reduction in layer 2/3 thickness was due to decreased cell numbers, we performed immunohistochemical staining using layer-specific markers. Satb2, which labels upper cortical neurons, was used to assess layer 2/3, while Tbr1, a marker for deeper layer 5/6 neurons, was used to evaluate potential differences in lower cortical layers (Figures 2D,H). DAPI staining was included in all the corresponding sections to visualize overall nuclear architecture. Across the three brains per genotype analyzed, we observed a 13.5% reduction in Satb2-positive cells in layer 2/3 of *Foxr1* knockout mice, although this difference did not reach statistical significance when averaged per animal (Figure 2E). However, when all brain sections were pooled by genotype, the reduction in Satb2-positive cells reached significance (*p* = 0.032, Figure 2F). Thus, while the average per-animal analysis showed a consistent trend, variability across animals reduced statistical power. Similarly, we found a ~14% decrease in DAPI-positive cells in layer 2/3 of *Foxr1* knockout mice compared to wildtype controls (*p* = 0.0501, Figure 2G). In contrast, no significant differences were observed in Tbr1-positive or DAPI-positive cell counts in the deeper cortical layers between wildtype and *Foxr1* knockout mice, consistent with the lack of changes in overall thickness of these layers (Figures 2I–K).

### 3.3 *Foxr1* deletion disrupts intermediate progenitor proliferation at E16.5

**Figure 3:**
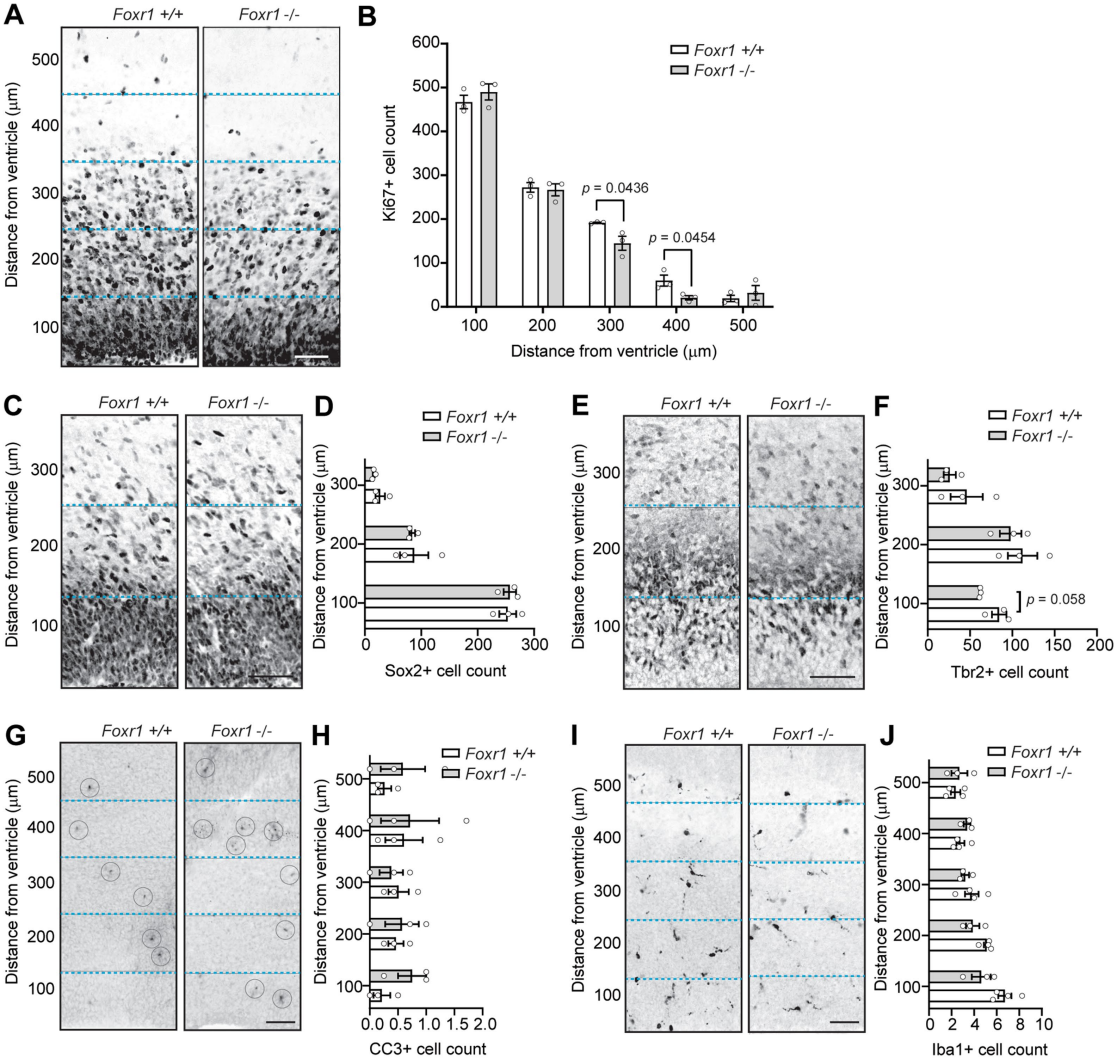
*Foxr1* knockout mice exhibit reduced intermediate progenitor proliferation at E16.5. **(A)** Representative Ki67 immunostaining in the cortical plate of *Foxr1* wildtype (+/+) and knockout (−/−) mice, marking proliferating cells. **(B)** Quantification of Ki67-positive cells in 100 μm bins from the ventricular surface shows a reduction in *Foxr1* −/− mice at 300 μm (*p* = 0.0436) and 400 μm (*p* = 0.0454). *N* = 3 animals per genotype; ~7 sections/animal. **(C)** Representative Sox2 immunostaining, labeling radial glial cells in the ventricular zone. **(D)** Quantification of Sox2-positive cells in 100 μm bins from ventricular surface shows no differences between genotypes. *N* = 3 animals per genotype; ~11 sections/animal. **(E)** Representative Tbr2 immunostaining, marking intermediate progenitors in the cortical plate. **(F)** Quantification of Tbr2-positive cells shows a reduction in the 0–100 μm bin from the ventricular surface in *Foxr1* −/− mice (*p* = 0.058). *N* = 3 animals per genotype; ~5 sections/animal. **(G)** Representative CC3 immunostaining showing apoptotic cells (circled). **(H)** Quantification of CC3-positive cells shows no difference between genotypes. *N* = 3 animals per genotype; ~6 sections/animal. **(I)** Representative Iba1 immunostaining, labeling microglia in the cortical plate. **(J)** Quantification of Iba1-positive cells shows no difference between genotypes. *N* = 3 animals per genotype; ~6 sections/animal. Dashed blue lines delineate 100 μm bins from the ventricular surface. Statistical significance was determined using unpaired Student’s *t*-tests. Error bars show SEM. All scale bars = 50 μm.

A smaller brain with fewer cells in layer 2/3 could result from premature depletion of the neural progenitor pool and/or increased apoptosis. To investigate potential changes in progenitor proliferation, we analyzed Ki67, a pan-proliferative marker that exhibits strong immunoreactivity adjacent the ventricular wall at E16.5 (Figure 3A). Quantification of Ki67-positive cells in 100 μm bins from the ventricular surface revealed a 24.5% reduction in proliferating cells in *Foxr1* knockout mice in the 300 μm bin and a 65.2% decrease in the 400 μm bin compared to wildtype controls (*p* = 0.0436 at 300 μm; *p* = 0.0454 at 400 μm, Figure 3B).

Next, we examined the distribution of two major progenitor cell populations in the embryonic *Foxr1* knockout telencephalon, defined by Sox2 and Tbr2 expression. As expected, Sox2 was highly expressed in radial glial cells, marking a major progenitor population closest to the ventricular zone, while Tbr2 labeled a more medial population of intermediate progenitor cells in the subventricular zone. At E16.5, we found no differences in Sox2-positive cell count between *Foxr1* knockout and wildtype telencephalon (Figures 3C,D), indicating that the radial glial progenitor pool remained intact. By contrast, quantification of Tbr2-positive cells revealed a 27% reduction in intermediate progenitors within the first 100 μm bin from the ventricular surface in *Foxr1* knockout telencephalon compared to wildtype control (*p* = 0.058, Figures 3E,F). These findings suggest that *Foxr1* deficiency selectively impacts the intermediate progenitor population while sparing radial glial cells.

Another potential contributor to reduced cortical size is increased cell death during early embryonic stages. To assess this, we performed immunohistochemical staining for cleaved caspase 3 (CC3), a marker of apoptosis, in the ventricular zone of *Foxr1* knockout brains at E16.5 (Figure 3G). Quantification of CC3-positive cells showed no differences between the *Foxr1* knockout and wildtype control telencephalon (Figure 3H). To determine whether perturbations to the E16.5 ventricular zone were associated with microglial activation, we stained for Iba1, a microglial marker (Figure 3I). Quantification of Iba1 revealed no changes in microglial presence in the nascent cortical column (Figure 3J).

### 3.4 Loss of *Foxr1* leads to hippocampal hypoplasia

**Figure 4:**
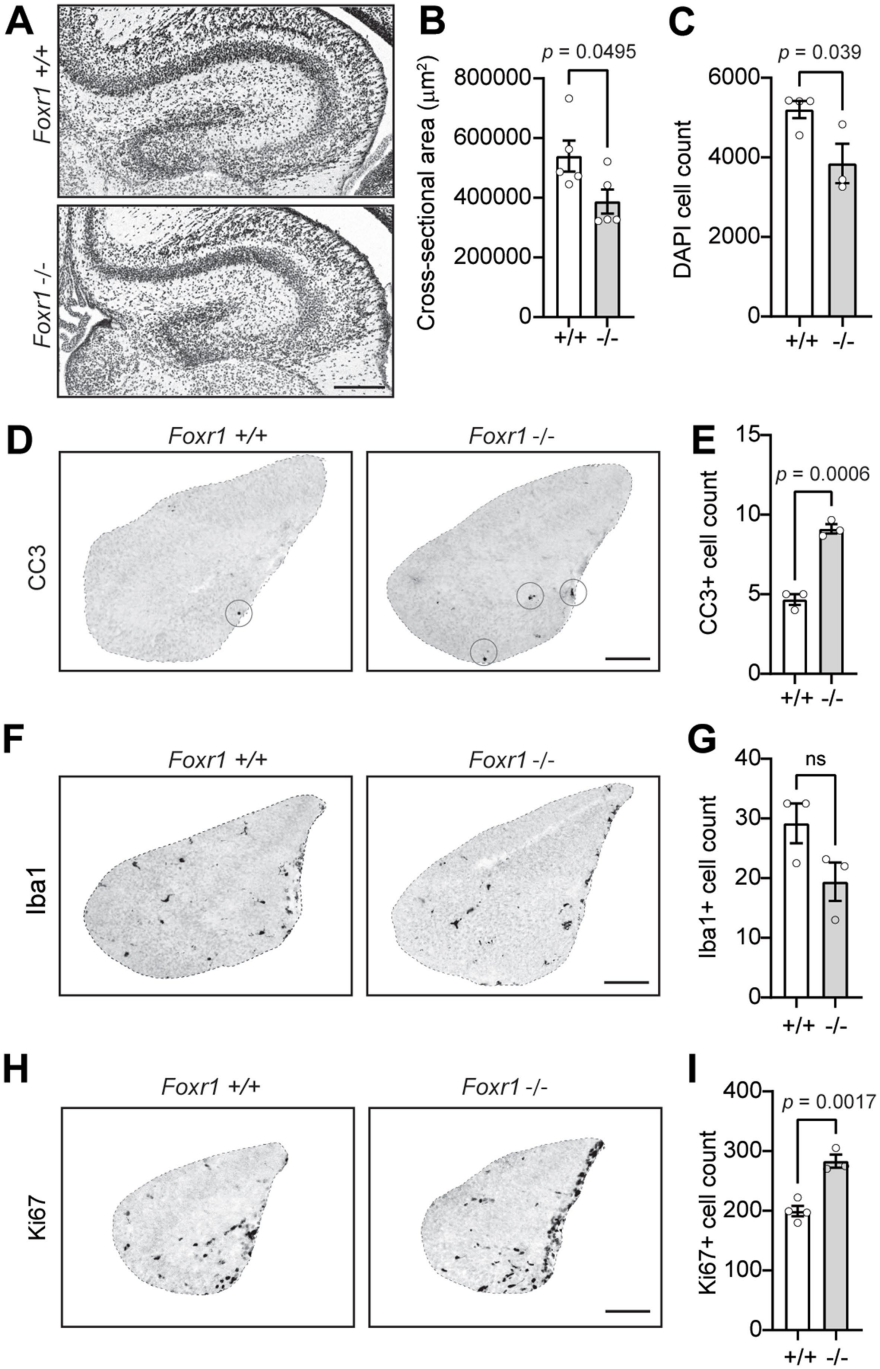
*Foxr1* knockout mice exhibit hippocampal hypoplasia, increased apoptosis, and altered progenitor proliferation. **(A)** Representative DAPI-stained coronal sections of the hippocampus from P0 *Foxr1* wildtype (+/+) and knockout (−/−) mice. Scale bar = 200 μm. **(B)** Quantification of hippocampal cross-sectional area shows a reduction in *Foxr1* −/− mice (*p* = 0.0495). *N* = 5 animals per genotype; ~4 sections/animal. **(C)** Quantification of DAPI-positive nuclei shows reduced total cell count in *Foxr1* −/− hippocampi (*p* = 0.039). *N* = 3 animals per genotype; ~6 sections/animal. **(D)** Representative CC3 immunostaining of coronal sections from E16.5 hippocampus, marking apoptotic cells (circled). Scale bar = 100 μm. **(E)** Quantification of CC3-positive cells shows a significant increase in apoptosis in *Foxr1* −/− hippocampi (*p* = 0.0006). *N* = 3 animals per genotype; ~3 sections/animal. **(F)** Representative Iba1 immunostaining of coronal sections from E16.5 hippocampus, labeling microglia. Scale bar = 100 μm. **(G)** Quantification of Iba1-positive cells show no difference between genotypes. *N* = 3 animals per genotype; ~4 sections/animal. **(H)** Representative Ki67 immunostaining of coronal sections from E16.5 hippocampus, labeling proliferating cells. Scale bar = 100 μm. **(I)** Quantification of Ki67-positive cells shows an increase in proliferation in *Foxr1* −/− hippocampi (*p* = 0.002). *N* = 4 mice (+/+), 3 mice (−/−); ~4 sections/animal. Statistical significance was determined using unpaired Student’s *t*-tests. Error bars indicate SEM.

Next, we investigated whether *Foxr1* is necessary for proper brain organization beyond the cerebral cortex. Nissl and DAPI staining of brain sections at P0 revealed a 28% reduction in hippocampal cross-sectional area in *Foxr1* knockout mice compared to wildtype controls (*p* = 0.0495, Figures 4A,B). This reduction is accompanied by a significant decrease in the number of DAPI-stained nuclei in the hippocampus of *Foxr1* knockout mice (26%, *p* = 0.039, Figure 4C), suggesting a loss of hippocampal cells.

To determine whether hippocampal hypoplasia in *Foxr1* knockout mice results from increased apoptosis, we examined CC3 staining in E16.5 hippocampal sections. We observed a 49% increase in CC3-positive cells in *Foxr1* knockouts compared to wildtype controls (*p* = 0.0006, Figures 4D,E). This increase in apoptosis was accompanied by a trending, though not statistically significant, reduction in Iba1-positive microglial counts in the hippocampus (Figures 4F,G). Notably, despite the elevated apoptosis, *Foxr1* knockout mice exhibited a significant 29.6% increase in Ki67-positive proliferating cells in the hippocampus at E16.5 (*p* = 0.0017, Figures 4H,I). We also examined additional brain regions, including the striatum, cerebellum, and corpus callosum, but found no notable differences between *Foxr1* knockouts and wildtype controls in these areas (Supplementary Figure 1).

## 4 Discussion

The study of Foxr1 function in brain development is limited by the high rate of embryonic lethality in *Foxr1* knockout mice, which restricts the generation of large experimental cohorts. Despite this constraint, our analysis of surviving knockout mice reveals that *Foxr1* loss results in microcephaly characterized by cortical thinning and hippocampal hypoplasia at birth. In the cortex, these structural deficits are primarily driven by impaired maintenance of neural progenitors, while in the hippocampus, hypoplasia is associated with increased apoptosis and dysregulated progenitor proliferation at E16.5.

Our findings demonstrate that cortical thinning in *Foxr1* knockout mice at P0 is restricted to the superficial layer 2/3, with no changes observed in deeper cortical layers. Alongside the reduction in thickness, we observed a trending decrease in Satb2-positive cell counts. Satb2, a transcription factor predominantly expressed in excitatory projection neurons, serves as a key marker of upper-layer cortical neurons. The comparable reduction in both DAPI- and Satb2-positive cells suggests that excitatory neuron likely underlies this deficit. This is supported by reduced numbers of Ki67- and Tbr2-positive intermediate progenitors at E16.5, with no change in Sox2-positive radial glial cells indicating a selective disruption in the progenitor population primarily responsible for generating post-mitotic excitatory neurons (Shen et al., 2006; Takahashi et al., 1999). As intermediate progenitors typically undergo symmetric divisions to produce upper-layer neurons (Kowalczyk et al., 2009), their reduction at E16.5 likely contributes to the reduced layer 2/3 population at P0 in *Foxr1* knockout mice. Given that layer 2/3 neurogenesis spans E16–18 (Polleux et al., 1997), our findings may capture the early onset of a broader developmental disruption.

The hippocampus, like the neocortex, is a laminated structure in which deeper layers form earlier and superficial layers emerge later (Cossart and Khazipov, 2022). Although hippocampal neurogenesis begins as early as E9 in mice, the timing of peak excitatory neuron production varies across its subregions: *Cornu ammonis* (CA) 2 neurons peak around E12.5, followed by CA3 at E14, CA1 at E15, and the dentate gyrus (DG) closer to birth (Bond et al., 2020). The termination of excitatory neurogenesis also occurs in a region-specific manner: CA2 concludes at E15, CA1 and CA3 by E16, and the DG continues into the postnatal period (P7–14) (Angevine, 1965; Bayer, 1980; Bond et al., 2020; Caviness, 1973). Therefore, the Ki67 immunoreactivity observed at E16.5 in the hippocampus likely reflects the final stages of neurogenesis in CA1 and CA3, and the early onset of neurogenesis in the DG.

Our findings of reduced hippocampal area and total cell counts at P0 suggest that *Foxr1* loss disrupts hippocampal neurogenesis, paralleling its effects in the cortex. The observed increase in Ki67-positive cells in the E16.5 *Foxr1* knockout hippocampus may reflect altered or prolonged progenitor proliferation, although this may be counterbalanced by the concurrent increase in apoptosis. Given that glutamatergic hippocampal neurons originate from the ventricular zone adjacent to the cortical hem (Cossart and Khazipov, 2022), Ki67 staining in this specific region would clarify whether progenitor proliferation is impaired. Future studies should also examine earlier time points (E12–E15.5), when CA neurogenesis peaks, to determine whether *Foxr1* disruption alters early progenitor dynamics. Additionally, assessing radial glia and intermediate progenitor populations in the hippocampus, as was done for the cortex, would help determine whether a shared mechanism underlies cortical and hippocampal deficits in *Foxr1* knockout mice.

Other brain regions examined, including the striatum, cerebellum, and corpus callosum, appear unaffected in *Foxr1* knockout mice at P0. However, the absence of early abnormalities does not preclude the emergence of postnatal phenotypes, given the extended developmental timelines of these structures. For example, the cerebellum undergoes granule cell proliferation from E12 through P30 (Consalez et al., 2021), with foliation beginning at E17.5 and continuing until P15. Similarly, the corpus callosum develops over an extended period, with axonal projections from cortical neurons emerging at E16 and extending into postnatal life (Chen et al., 2017). Development of the corpus callosum is highly activity-dependent, with refinement of interhemispheric connections extending through at least P30 (Zhou et al., 2013). Notably, since callosal projection neurons primarily arise from cortical layers 2/3 and 5 (Zhou et al., 2013), the reduction in layer 2/3 neurons observed at P0 in *Foxr1* knockout mice may predispose mice to delayed or impaired callosal development. Although we did not detect structural differences in the corpus callosum at P0 in *Foxr1* knockout mice, future studies are needed to determine whether morphological or functional deficits emerge later in development.

Our findings establish Foxr1 as a critical regulator of progenitor cell dynamics and neuronal production during cortical and hippocampal development. *Foxr1* knockout mice exhibit microcephaly characterized by cortical thinning and hippocampal hypoplasia. In the cortex, this is driven by impaired intermediate progenitor proliferation, particularly affecting upper-layer neuron formation. In the hippocampus, hypoplasia may result from a combination of increased apoptosis and disrupted neurogenesis. These region- and layer-specific effects underscore the essential role of Foxr1 in brain development and have broader implications for understanding the molecular underpinnings of microcephaly and related neurodevelopmental disorders.

## Data Availability

The raw data supporting the conclusions of this article will be made available by the authors, without undue reservation.

## Supplementary material

SUPPLEMENTARY FIGURE 1 *Foxr1* knockout does not affect striatum, cerebellum or corpus callosum development at P0. **(A)** Representative Ctip2-stained coronal brain sections from *Foxr1* wildtype (+/+) and knockout (−/−) mice, spanning the rostral to caudal regions of the striatum. **(B)** Quantification of total cross-sectional striatal area shows no differences between genotypes. *N* = 5 animals (*+*/*+*) and 4 animals (−/−); ~4 sections/animal. **(C)** Representative DAPI-stained sagittal cerebellar sections from *Foxr1* +/+ and −/− mice. **(D)** Quantification of cerebellar cross-sectional area reveals no differences between genotypes. *N* = 3 animals per genotype; ~6 sections/animal. **(E)** Representative Map2 immunostaining of coronal sections showing measurements of cortical thickness (yellow line) and lateral corpus callosum thickness (black line). **(F)** Quantification of lateral corpus callosum thickness shows no difference between genotypes. *N* = 4 animals per genotype; ~5 sections/animal. **(G)** Representative Tbr1 immunostaining of coronal sections showing medial corpus callosum thickness (black line). **(H)** Quantification of medial corpus callosum thickness showing no differences between genotypes. *N* = 3 animals (+/+) and 4 animals (−/−); ~6 sections/animal. Scale bars = 200 μm for **(A,C,E)** and 50 μm for **(G)**. Statistical significance was determined using unpaired Student’s *t*-tests. Error bars indicate SEM.

SUPPLEMENTARY TABLE 1 Primary and secondary antibodies used for immunostaining.
