# Supplementary figures and images for: Foxr1 deletion causes microcephaly and leads to cortical and hippocampal hypoplasia
Source: Front Neurosci. 2025 May 27;19:1589043. doi: 10.3389/fnins.2025.1589043 (PMC12149149; doi:10.3389/fnins.2025.1589043)

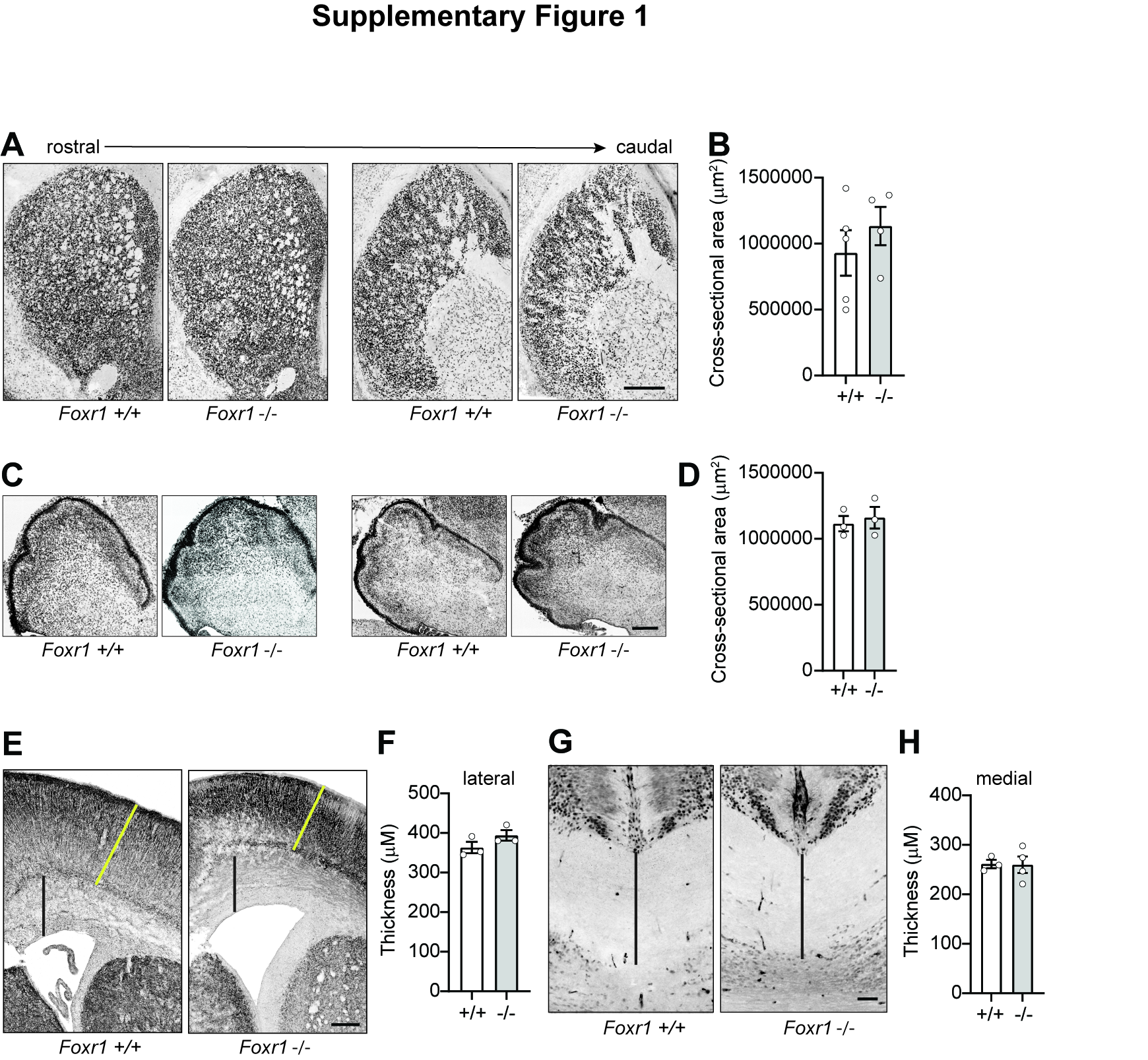

Supplement: SUPPLEMENTARY FIGURE 1 — Foxr1 knockout does not affect striatum, cerebellum or corpus callosum development at P0. (A) Representative Ctip2-stained coronal brain sections from Foxr1 wildtype (+/+) and knockout (−/−) mice, spanning the rostral to caudal regions of the striatum. (B) Quantification of total cross-sectional striatal area shows no differences between genotypes. N = 5 animals (+/+) and 4 animals (−/−); ~4 sections/animal. (C) Representative DAPI-stained sagittal cerebellar sections from Foxr1 +/+ and −/− mice. (D) Quantification of cerebellar cross-sectional area reveals no differences between genotypes. N = 3 animals per genotype; ~6 sections/animal. (E) Representative Map2 immunostaining of coronal sections showing measurements of cortical thickness (yellow line) and lateral corpus callosum thickness (black line). (F) Quantification of lateral corpus callosum thickness shows no difference between genotypes. N = 4 animals per genotype; ~5 sections/animal. (G) Representative Tbr1 immunostaining of coronal sections showing medial corpus callosum thickness (black line). (H) Quantification of medial corpus callosum thickness showing no differences between genotypes. N = 3 animals (+/+) and 4 animals (−/−); ~6 sections/animal. Scale bars = 200 μm for (A,C,E) and 50 μm for (G). Statistical significance was determined using unpaired Student’s t-tests. Error bars indicate SEM. [file Image_1.tif]
